# Plant resistance against whitefly and its engineering

**DOI:** 10.3389/fpls.2023.1232735

**Published:** 2023-08-30

**Authors:** Di Li, Heng-Yu Li, Jing-Ru Zhang, Yi-Jie Wu, Shi-Xing Zhao, Shu-Sheng Liu, Li-Long Pan

**Affiliations:** ^1^ Ministry of Agriculture Key Laboratory of Molecular Biology of Crop Pathogens and Insects, Key Laboratory of Biology of Crop Pathogens and Insects of Zhejiang Province, Institute of Insect Sciences, Zhejiang University, Hangzhou, China; ^2^ The Rural Development Academy, Zhejiang University, Hangzhou, China

**Keywords:** phloem-feeding insects, *Bemisia tabaci*, plant defense, plant-whitefly interaction, resistance breeding

## Abstract

Plants face constant threats from insect herbivores, which limit plant distribution and abundance in nature and crop productivity in agricultural ecosystems. In recent decades, the whitefly *Bemisia tabaci*, a group of phloem-feeding insects, has emerged as pests of global significance. In this article, we summarize current knowledge on plant defenses against whitefly and approaches to engineer plant resistance to whitefly. Physically, plants deploy trichome and acylsugar-based strategies to restrain nutrient extraction by whitefly. Chemically, toxic secondary metabolites such as terpenoids confer resistance against whitefly in plants. Moreover, the jasmonate (JA) signaling pathway seems to be the major regulator of whitefly resistance in many plants. We next review advances in interfering with whitefly-plant interface by engineering of plant resistance using conventional and biotechnology-based breeding. These breeding programs have yielded many plant lines with high resistance against whitefly, which hold promises for whitefly control in the field. Finally, we conclude with an outlook on several issues of particular relevance to the nature and engineering of plant resistance against whitefly.

## 1 Introduction

Plants, whether wild or cultivated, are constantly confronted with many biotic and abiotic threats (Wilkinson et al., 2019). The biotic threats encompass pathogens such as viruses, bacteria and fungi, as well as herbivores including insects and large animals. Among these biotic factors, insect herbivores are particularly significant due to their remarkable diversity and abundance (Savary et al., 2019; Wilkinson et al., 2019). Extensive research in recent decades has revealed general principles underlying the intimate interactions between insect herbivores and their plant hosts (Erb and Reymond, 2019; Snoeck et al., 2022). Insect herbivores employ a range of behavioral and molecular strategies to facilitate nutrient extraction (Dussourd, 2017; Stahl et al., 2018), while plants deploy various defense responses to deter insect herbivores (Erb and Reymond, 2019; Snoeck et al., 2022). The long-lasting and ongoing arms race between plants and insect herbivores has shaped the ecology and evolution of both groups of organisms in nature (Bergelson et al., 2001; Zust et al., 2012). In agricultural practices, insect herbivores, alongside other pests, pose serious threats to global food security (Savary et al., 2019). Therefore, an improved understanding of plant-insect herbivore interactions is crucial, from both scientific and applied perspectives.

Many insect herbivores such as the whitefly, have specialized in feeding on plant phloem and thus are referred to as phloem-feeding insects. Despite their small size (around 1.0 mm in length for adults), whiteflies are highly prolific, with females each capable of producing dozens to hundreds of eggs depending on environmental conditions (Byrne and Bellows, 1991). Whiteflies cause significant losses to crop through direct sap feeding, inducing plant physiological disorders and promoting the growth of sooty mold (Oliveira et al., 2001; Farina et al., 2022). Moreover, whiteflies can indirectly harm plants by transmitting plant viruses, particularly begomoviruses and criniviruses, resulting in severe viral disease epidemics (Gilbertson et al., 2015; Fiallo-Olivé et al., 2020; Wang and Blanc, 2021). For example, whiteflies are known to vector over 400 viruses belonging to the genus *Begomovirus* through a persistent circulative manner, leading to the occurrence of numerous viral diseases (Gilbertson et al., 2015; Fiallo-Olivé et al., 2020; Wang and Blanc, 2021; Fiallo-Olivé and Navas-Castillo, 2023).

As a group of piercing-sucking insects, the feeding behavior of whitefly differs significantly from that of insects with chewing mouthparts. Correspondingly, the responses of plants to whitefly infestation differ significantly from those mounted against chewing insects (Kaloshian and Walling, 2005; Kempema et al., 2007; Zarate et al., 2007; Walling, 2008; Wang et al., 2017). Additionally, when compared to some other closely-related piercing-sucking insects including aphids, whiteflies are unique in many ways with regard to interactions with plants due to their distinctive size, feeding habits and life history (Walling, 2008; Wang et al., 2017). Therefore, explorations of plant resistance against whitefly may add to our knowledge of insect-plant interactions. More importantly, the distinctiveness of whitefly-plant interactions urges more efforts in resistance engineering as plant cultivars obtained from breeding programs against other groups of insect herbivores may fail to control whitefly. Under this scenario, innovations to specifically augment plant resistance against whitefly are required and will be invaluable in sustaining the production of whitefly-susceptible crops.

The continuous research efforts and rapid development of novel research tools such as omics, have promoted the dissection of plant resistance against whitefly (Zogli et al., 2020). Additionally, in recent years, significant advances have been made in interfering with the whitefly-plant interface through the engineering of plant resistance. In this article, we aim to summarize and review these recent advances. First, we will describe current understanding of plant traits that confer resistance to whitefly. Next, we will summarize the progress made in engineering plant resistance to whitefly using conventional and biotechnology-based breeding. Finally, we will highlight several issues related to the investigation and engineering of plant resistance against whitefly.

## 2 Plant physical traits that confer resistance to whitefly

**Figure 1:**
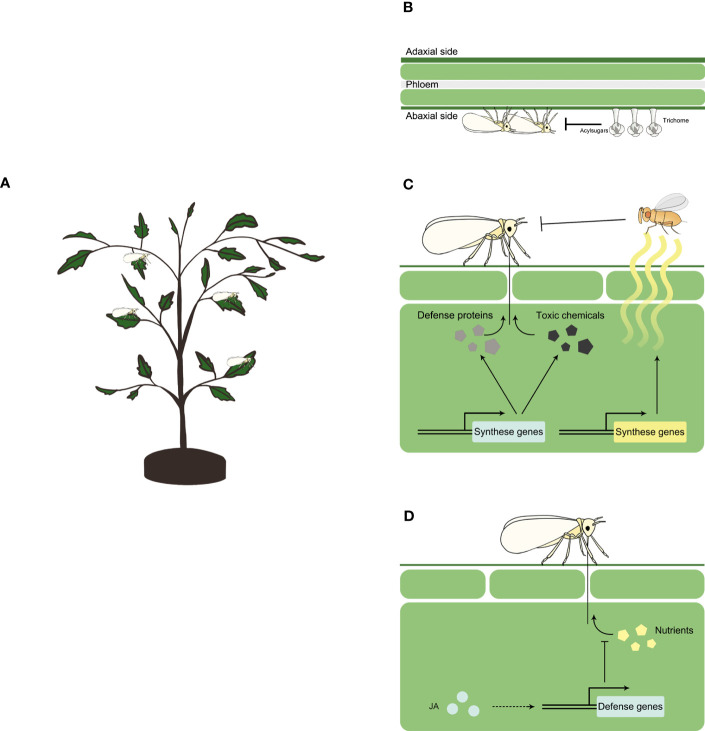
Plant resistance against whitefly Schematic representation of whitefly feeding a plant **(A)**, and plant resistance against whitefly at physical **(B)**, chemical **(C)** and signaling **(D)** level. Physically, plants may use trichome and acylsugar to constrain whitefly feeding. Chemically, plants may synthase a repertoire of secondary metabolites such as terpenoids, glucosinolates, phenolic compounds and lignin, and defense proteins such as glucosidase, glucanase and chitinase to inhibit whitefly herbivory. Plants may also synthase and release volatile organic chemicals (VOCs) such as ocimene, myrcene, methyl salicylate and tetradecane, to attract natural enemies of whitefly. At signaling level, jasmonates (JA) controls the expression of defense genes to inhibit the ingestion of plant nutrients by whitefly.

Physical traits of resistance impact insect herbivores physically, such as restricting their movement or hindering their feeding. When whiteflies feed on plants (**Figure 1A**), trichomes and acylsugars serve as major physical traits that confer resistance to whitefly (**Figure 1B**).

### 2.1 Trichomes

Trichomes are specialized hairs on the surface of plants that can be epidermal protuberances of different sizes, shapes and arrangements. They can be classified as glandular and non-glandular based on their ability to synthesize, secrete and store substances (Tissier, 2012). The role of trichomes in whitefly-plant interactions has been intensively studied in wild relatives of cultivated tomato, in which trichomes were categorized into seven types with four of them being glandular (types I, IV, VI, and VII) and three being non-glandular (types II, III, and V) (Simmons and Gurr, 2005). Glandular trichomes and their exudates play an important role in plant defense against whitefly (**Figure 1B**). The entrapment of whitefly on tomato leaves was first reported by Kisha (1981). Further exploration revealed a key role of glandular type IV and VI trichomes in reducing whitefly adult survival and oviposition rate (Channarayappa et al., 1992; Snyder et al., 1998). Detailed profiling of whitefly feeding activities revealed that type IV glandular trichomes disrupted whitefly probing behavior (Narita et al., 2023).

Compounds in the exudates of these trichomes such as acylsugars were identified to be vital in conferring resistance to whitefly in plants including tomato and *Nicotiana benthamiana*. High resistance against whitefly was mechanically transferable by applying the trichome exudates from resistant *S. pennellii* accessions (LA716, LA1340, LA1674 and LA2560) onto the leaves of susceptible tomato plants (Liedl et al., 1995; Muigai et al., 2002). Significant, positive correlations were found between acylsugar content and whitefly resistance when analyzing tomato genotypes with varying acylsugar contents (Dias et al., 2016; Marchant et al., 2020; Dias et al., 2021; de Lima Filho et al., 2022). Furthermore, acylsugar compositions from several *S. pennellii* accessions with high whitefly resistance were characterized, revealing synergistic interactions between different kinds of acylsugars (Leckie et al., 2016). In *N. benthamiana* plants, CRISPR/Cas9 mutagenesis of acylsugar acyltransferase genes significantly decreased acylsugar contents and resistance against whitefly while maintaining the structure and abundance of trichomes on the leaf surface (Feng et al., 2022).

The role of trichomes in other plants has also been explored. Many studies analyzed the correlation between whitefly resistance and overall trichome density without classifying trichomes into specific types such as glandular or non-glandular. Results have shown that the role of trichomes varies depending on the plant species, highlighting intrinsic variation between plant species. For example, in tobacco and cassava, negative correlation was observed between whitefly resistance and overall trichome density (Li et al., 2014; Pastório et al., 2023), whereas in black gram a positive correlation was found (Taggar and Gill, 2012). On the other hand, no significant correlation was identified between whitefly resistance and trichome density in cucumber (Novaes et al., 2020). In some cases, the correlation may vary among cultivars of the same plant species. Field trials on cotton cultivars, for example, showed higher whitefly population density on cotton plants with higher trichome density, indicating a negative correlation between trichome density and whitefly resistance (Zia et al., 2011; Prado et al., 2016; Siddiqui et al., 2021; Suthar et al., 2022). However, positive correlations between overall trichome density and whitefly resistance in cotton have also been reported (Thomas et al., 2014; Zhu et al., 2018). Similar variations have been observed in studies on soybean (McAuslane, 1996; Baldin et al., 2017). Furthermore, the contribution of trichome length to plant defenses against whitefly has been investigated, revealing negative correlation between trichome length and plant defenses against whitefly in eggplant and black gram plants (Taggar and Gill, 2012; Hasanuzzaman et al., 2016).

So far, studies conducted on tomato indicate that trichomes exhibiting defenses against whitefly have been observed only in wild relatives of cultivated tomato and tomato cultivars obtained from breeding programs involving wild relatives of tomato. For other plant species, studies have been mostly focused on crop cultivars, and the results generally suggest a lack of contribution of trichomes to plant defense. To clarify the role of trichomes in plant defenses against whitefly in plant species beyond tomato, it is necessary to investigate the trichomes of wild relatives of these crop species. Furthermore, in studies involving non-tomato plants, the correlation between whitefly resistance and overall trichome density is often analyzed. However, this approach may mask the function of specific trichome types, such as glandular trichomes, which play a significant role in whitefly resistance. Therefore, further empirical studies are needed to gain a better understanding on the role of trichomes in plant defense against whitefly.

### 2.2 Other physical traits

In addition to trichomes, other physical traits such as leaf shape, color and lamina thickness have been investigated for their potential contribution to plant defenses against whitefly. For example, cotton varieties with okra-shaped leaves exhibited higher whitefly resistance compared to broad-leaved varieties (Chu et al., 2002). Narrow and thinner leaves were associated with increased whitefly resistance in tomato breeding lines (Pal et al., 2021). Leaf color also plays a role, as eggplant varieties with leaves reflecting more green light and exhibiting higher overall brightness displayed higher whitefly resistance (Hasanuzzaman et al., 2016). Conversely, in common bean, luminosity and the intensity of green and yellow colors were negatively correlated with whitefly resistance (Santos et al., 2020). Regarding leaf lamina thickness, field tests involving green gram, cotton, cucumber and eggplant, consistently reported higher whitefly populations on varieties with thicker leaf lamina, indicating a negative correlation between leaf lamina thickness and whitefly resistance (Butter and Vir, 1989; Shibuya et al., 2009; Jindal and Dhaliwal, 2011; Hasanuzzaman et al., 2016). Although these morphological traits have been implicated in contributing to resistance in many studies, their precise roles in whitefly-plant interactions have yet to be determined. Further detailed investigations are necessary to dissect the specific functions of these traits, categorize them and consider them in resistance breeding programs.

## 3 Plant chemical traits that confer resistance to whitefly

In plant resistance against insect herbivores, plant chemicals play a significant role either directly or indirectly by attracting natural enemies of the herbivores, thereby providing protection to the plants (Yactayo-Chang et al., 2020). In the context of whitefly-plant interactions, several chemicals that contribute to direct or indirect defenses against whitefly have been identified (**Figure 1C**).

### 3.1 Direct chemical defense

#### 3.1.1 Secondary metabolites

Secondary metabolites play a major role in plant defense against insect herbivores (Luo et al., 2023), yet only a few have been examined for their contribution to resistance against whitefly. Luan et al. (2013) found that whitefly feeding increased the contents of several terpenoids including cedinene in tobacco plants, and manipulation of cedinene contents through gene silencing or over-expression of *5-epi-aristolochene synthase* indicated that cedinene positively regulated resistance against whitefly. As revealed by metabolites profiling and feeding assays, many phenolic glycosides from tomato plants were identified to contribute to plant defenses against whitefly (Xia et al., 2021). Glucosinolates are a major group of secondary metabolites in crucifers, and are shown to contribute to resistance against whitefly when they accumulated to unnaturally high levels, but not under natural conditions. Elbaz et al. (2012) found that the survival and developmental rate of whitefly nymphs significantly decreased with the accumulation of aliphatic glucosinolate through *AtMYB29* overexpression. Whitefly oviposition preference for *Arabidopsis thaliana* plants was significantly reduced when the contents of aliphatic and total glucosinolates were increased to unnaturally high levels through *AtMYB28* and *AtMYB51* over-expression (Markovich et al., 2013). Using *Brassica* crops that vary in glucosinolate profile and *Arabidopsis* mutants defective in glucosinolate biosynthesis or hydrolysis, Li et al. (2021) revealed that the performance of invasive MEAM1 whiteflies and indigenous Asia II 3 whiteflies was unaffected by glucosinolates when these chemicals were maintained at natural levels in these plants.

Additionally, other secondary metabolites have been implicated in plant defense against whitefly. Studies have reported a positive correlation between the total content of phenolic components and whitefly resistance in eggplant and tomato (Hasanuzzaman et al., 2016; Pal et al., 2021). In tobacco plants, it was observed that certain phenolic compounds, such as chlorogenic acid, catechin, cafeic acid, p-coumaric acid, rutin and ferulic acid, increased in response to whitefly infestations, suggesting their potential role in resistance (Zhang et al., 2017). Similarly, in soybean and cassava, rutin and lignin (along with its derivatives) showed positive association with whitefly resistance (Vieira et al., 2016; Perez-Fons et al., 2019). Furthermore, whitefly infestation has been found to induce callose deposition, which may contribute to plant defense by plugging sieve pores (Kempema et al., 2007; Li et al., 2017).

Many plant secondary metabolites have been shown empirically to contribute to plant defense against insects with chewing mouthparts (Yactayo-Chang et al., 2020; Luo et al., 2023). However, research on the contribution of secondary metabolites to plant defense against whitefly has been limited. There are still many unanswered questions regarding the induction and mechanisms of action of secondary metabolites in whitefly defense. To address these gaps, it would be valuable to draw upon the abundant information available from studies on chewing insects (Yactayo-Chang et al., 2020; Luo et al., 2023). A better understanding of how plant secondary metabolites function in whitefly resistance could have practical implications for the development of novel pesticides.

#### 3.1.2 Defense proteins

Upon infestation by insect herbivores, plants can activate the expression of defense proteins, which can disrupt the normal physiological processes of the insects, including digestion and absorption of nutrients (Howe and Jander, 2008). The role of defense proteins in resistance against whitefly has been extensively studied, particularly focusing on CYS6, a protease inhibitor in tobacco plants. Genetic manipulation of *CYS6* and feeding assays using purified CYS6 have demonstrated its direct contribution to plant defense against whitefly (Du et al., 2022). Additionally, whitefly infestation has been found to induce the expression of various plant defense proteins. For example, increased local expression of *β-glucosidase* was found in squash plants infested by whitefly (van de Ven et al., 2000). Tomato and cassava plants, when infested by whitefly, showed significant increases in the expression of *β-1,3-glucanase*, *chitinase* and *peroxidase* (Mayer et al., 1996; Antony and Palaniswami, 2006). Furthermore, whitefly infestation led to increased activity of polyphenoloxidase in cucumber and pepper plants, as well as superoxide dismutase, peroxidase and polyphenoloxidase in tomato and soybean plants (Zhang et al., 2008; Latournerie-Moreno et al., 2015; de Lima Toledo et al., 2021; Harish et al., 2023). Additionally, the expression of several pathogenesis-related proteins in tomato was also found to be upregulated in response to whitefly infestation (Puthoff et al., 2010).

The accumulation of defense proteins in response to whitefly infestation is widely acknowledged, but their precious role in plant defenses against whitefly remains largely unknown. To address this knowledge gap, additional case studies, similar to the work of Du et al. (2022) are necessary. Assays involving genetic manipulation of plant genes that encode whitefly infestation-inducible defense proteins and feeding experiment with purified proteins would provide valuable insights into the genuine contribution of these defense proteins to resistance against whitefly.

### 3.2 Indirect defenses

In addition to direct defense, whitefly feeding can induce the release of plant volatile organic compounds (VOCs) in plants, which attract the natural enemies of whitefly and help protect the plants (**Figure 1C**). In terms of predators, Nomikou et al. (2005) found that two predatory mites *Typhlodromips swirskii* and *Euseius scutalis* showed a significant preference for whitefly-infested cucumber plants compared to non-infested plants, and this preference was mediated by the volatiles emitted by plants. Silva et al. (2018) showed that the predatory mirid *Macrolophus basicornis* was attracted to tomato plants infested by a mixture of whitefly eggs, nymphs and adults. As for parasitoids, Zhang et al. (2013) demonstrated that whitefly infestation in *Arabidopsis* plants led to the accumulation of ocimene/myrcene, which effectively attracted the whitefly parasitoid *Encarsia formosa*. In response to whitefly herbivory, melon plants released methyl salicylate and tetradecane, which facilitated the attraction of the whitefly parasitoid *E. desantisi* (Silveira et al., 2018). Similarly, whitefly infestation of tomato plants resulted in the emission of β-myrcene and β-caryophyllene, which mediated host location of the parasitic wasp *E. formosa* (Chen et al., 2020).

Due to the differences in feeding behavior, the quantity and quality of VOCs produced by whitefly-infested plants are expected to vary compared to plants attacked by other insects. For example, whitefly may interfere with the indirect plant defense mounted against spider mites in Lima bean (Zhang et al., 2009). It should be noted, however, whitefly-induced VOCs have been identified only in a few case studies (see above). In addition to VOCs, other plant traits such as architecture and glandular trichomes also contribute to indirect defenses against insect herbivores (Pearse et al., 2020). Therefore, further research is required to fully explore the potential of indirect defenses in whitefly control, by investigating plant characteristics that facilitate natural enemy-mediated plant protection.

## 4 Plant signaling pathway against whitefly

The jasmonate (JA) signaling pathway, as a conserved core pathway regulating plant response to insect herbivory (Erb and Reymond, 2019), plays a major role in plant defense against whitefly (**Figure 1D**). Studies using *Arabidopsis* mutants with varying levels of JA defense have demonstrated the control of basal defense against whitefly by JA signaling pathway (Zarate et al., 2007). Manipulation of JA signaling pathways in tobacco plants through virus-induced gene silencing or genetic mutation of *MYC2* resulted in increased whitefly survival and fecundity (Zhang et al., 2012; Li et al., 2014). In tomato, when compared to control, whitefly survival and fecundity increased on JA-deficient *spr2* mutant plants and decreased on JA-overexpression 35S-*prosystemin* transgenic plants (Sun et al., 2017). Exogenous application of JA on tomato plants significantly reduced whitefly survival and fecundity (Shi et al., 2017). Additionally, several downstream defense genes involved in JA signaling pathway against whitefly have been identified, including terpenoid synthesis genes, the expression of which is positively modulated by JA treatment (Li et al., 2014).

## 5 Engineering of plant resistance to whitefly

Both conventional and biotechnology-based breeding approaches have been employed in the engineering of plant resistance against whitefly. These research endeavors have resulted in the development of numerous genetic resources that can be utilized to enhance plant resistance against whitefly.

### 5.1 Naturally occurring resistances and their utilization in resistance breeding

Whiteflies exhibit variability in their host plant range, with different species showing variations in survival and fecundity on different plant species, as well as cultivars or ecotypes of the same plant species (Zang et al., 2006; Xu et al., 2011). Wild relatives of cultivated crops often exhibit higher resistance to insect pests compared to crop cultivars (Li et al., 2018; Ferrero et al., 2020). The naturally occurring resistance found in these plants, including genes or quantitative trait loci (QTLs) associated with resistance, can be directly utilized in resistance breeding (Broekgaarden et al., 2011). Consequently, significant research efforts have been dedicated to the identification of plant resistance genes or QTLs and their application in breeding initiatives.

#### 5.1.1 Plant resistance genes or QTLs conferring resistance to whitefly

Tomato has been extensively studied in the context of resistance genes or QTLs due to the significance of whitefly in tomato production. Several whitefly resistance genes or QTLs have been identified in tomato, providing valuable genetic resources for breeding. Among these genes, *Mi-1* has been the focus of extensive research (Nombela and Muñiz, 2010). *Mi-1*, a member of nucleotide-binding, leucine-rich repeat family of resistance genes, was initially identified for conferring resistance to root-knot nematodes (Roberts and Thomason, 1986). It was later found to also impart resistance to phloem-feeding insects like aphids and whiteflies (Rossi et al., 1998; Nombela et al., 2003). Subsequent analysis revealed a series of plant factors that may affect whitefly resistance conferred by *Mi-1*, such as Hsp90, salicylic acid and plant age and size (Rodríguez-Álvarez et al., 2015; Rodríguez-Álvarez et al., 2017; Pascual et al., 2023). Additionally, functional characterization of *Mi-1.2-*like orthologs in cotton demonstrated their potential contribution to plant resistance against whitefly, highlighting the potential of *Mi-1.2*-like genes in whitefly control in cotton plants (Aslam et al., 2023). Furthermore, several other genes or QTLs that may confer whitefly resistance, such as *Wf-1* and *Wf-2*, have been identified in *S. pennellii*, *S. galapagense* and *S. habrochaites* (Leckie et al., 2012; Firdaus et al., 2013; Lucatti et al., 2014; Santegoets et al., 2021). Notably, a major QTL that controls the density of type IV trichomes, a major contributor to whitefly resistance, was identified in *S. pimpinellifolium* (Mata-Nicolás et al., 2021).

In contrast, in other crops and their wild relatives, the identification of QTLs related to whitefly resistance is limited, indicating the need for further research. For example, in melon, only two additive QTLs affecting whitefly fecundity, namely *BtB-VII.1* and *BtB-IX.1*, have been identified (Boissot et al., 2010). In soybean, whitefly resistance was found to be controlled by two major genes as well as polygenes, with the major genes showing an inheritability of over 85% (Xu et al., 2010).

#### 5.1.2 Conventional resistance breeding

Conventional breeding for plant resistance involves incorporating resistance traits from highly resistant plant accessions into target crop cultivars. So far, conventional resistance breeding has been reported only in tomato. In the first attempt, a tomato cultivar carrying the *Mi-1* gene, Motelle, was obtained from the crossing between *S. lycopersicum* Moneymaker and *S. peruvianum*; detailed mapping revealed that Motelle differed from Moneymaker only in the presence of a 650 kb region containing the *Mi-1* gene from *S. peruvianum* (Ho et al., 1992). While Motelle was initially obtained for nematode control, subsequent studies revealed that plants of this cultivar displayed significantly lower susceptibility to whitefly than Moneymaker (Nombela et al., 2000; Jiang et al., 2001). Since then, several more studies have been reported using wild relatives of cultivated tomato as donor of whitefly resistance. For example, plant traits associated with whitefly resistance from the wild tomato *S. pimpinellifolium* accession TO-937 were introgressed into Moneymaker, resulting in lines with increased whitefly resistance (Rodríguez-López et al., 2011; Escobar-Bravo et al., 2016). Several mini tomato lines that displayed high whitefly resistance were obtained through interspecific crossing between *S. lycopersicum* mini tomato cultivars and *S. pennellii* LA-716 (Maciel et al., 2017). Tomato lines obtained from the above attempts were further used in resistance breeding. For example, Gouveia et al. (2018) used two tomato lines that carry the *Mi* gene and differ in acyl-sugar content in a crossing experiment and obtained several tomato lines with high whitefly resistance.

It is important to note that tomato cultivars obtained through conventional resistance breeding, using wild *Solanum* species as donors of whitefly resistance, exhibit only partial resistance against whitefly. This may be attributed to the fact that whiteflies are capable of surviving and reproducing on wild relatives of crops, albeit with reduced performance compared to that on cultivated crops. Moreover, conventional resistance breeding is characterized by its unpredictable nature and time-consuming processes, underscoring the need for biotechnology-based approaches in whitefly resistance breeding.

### 5.2 Biotechnology-based resistance breeding

Biotechnology-based breeding, which entails targeted manipulation or introduction of genetic materials in crops, represents a promising alternative for crop breeding as it is more targeted and efficient (Barrows et al., 2014). In biotechnology-based resistance breeding against whitefly, plant-mediated RNA interference (RNAi) of whitefly genes and ectopic expression of insecticidal proteins or foreign genes that manipulate the production of insecticidal chemicals in plants, have been explored.

#### 5.2.1 Plant-mediated RNA interference of whitefly genes

RNAi, a specific post-transcriptional gene silencing mechanism triggered by double-stranded RNA (dsRNA) or small interfering RNA (siRNA), has been harnessed in resistance breeding for whitefly management (**Table 1**). Ghanim et al. (2007) demonstrated that injection of dsRNA into whitefly hemolymph activated RNAi and downregulated of the transcription of target genes, confirming the presence and functionality of the RNAi machinery. Subsequently, the efficacy of orally-delivered dsRNAs/siRNAs was compared, showing that targeting the *V-ATPase A* led to efficient downregulation of the target gene and high whitefly mortality (Upadhyay et al., 2011). Based on these findings, transgenic tobacco plants were generated to produce long dsRNA precursor that would produce siRNAs targeting whitefly *V-ATPase A* mRNA. Bioassay revealed that expressing the dsRNA precursor significantly downregulated *V*-*ATPase A* transcription, increased whitefly mortality, and protected tobacco plants from heavy whitefly infestation (Thakur et al., 2014). Similarly, the expression of dsRNA in lettuce or siRNA in common beans and tomato targeting a whitefly *V-ATPase* gene, significantly increased resistance to whitefly (Ibrahim et al., 2017; Ferreira et al., 2022; Pizetta et al., 2022). Additional studies have employed RNAi to downregulate whitefly genes, highlighting the promising potential of this approach in whitefly management (Malik et al., 2016; Eakteiman et al., 2018). The expression of dsRNA targeting whitefly *acetylcholinesterase*, *ecdysone receptor* and two *trehalose-6-phosphate synthase genes* in tobacco plants conferred resistance against whitefly (Malik et al., 2016; Gong et al., 2022).

**Table 1:** Resistance engineering in plants that targets whitefly genes using RNA interference.

Target genes in whitefly	Methods of targeting	Test plant	Effects on whitefly	Reference
*v-ATPase A*	Expression of dsRNA	*Nicotiana tabacum*	Decreased adult survival	Thakur et al., 2014
*Acetylcholinesterase* and *ecdysone receptor*	Expression of dsRNA	*N*. *tabacum*	Decreased adult survival	Malik et al., 2016
*v-ATPase*	Expression of dsRNA	*Lactuca sativa*	Decreased adult survival and fecundity	Ibrahim et al., 2017
*F1F0 ATP synthases*	Expression of miRNA	*Gossypium hirsutum*	Decreased adult survival	Wamiq and Khan, 2018
*Sex lethal*, *acetylcholinesterase* and *orcokinin*	Expression of artificial miRNA	*N*. *tabacum*	Retarded nymph development, decreased population growth	Zubair et al., 2020
*v-ATPase*	Expression of siRNA	*Phaseolus vulgaris*	Decreased adult survival	Ferreira et al., 2022
*Trehalose-6-phosphate synthase 1* and *2*	Expression of dsRNA	*N*. *tabacum*	Retarded nymph development, decreased adult survival and fecundity	Gong et al., 2022
*v-ATPase A*	Expression of siRNA	*Solanum lycopersicum*	Decreased adult survival and fecundity	Pizetta et al., 2022
*Aquaporin* and *alpha glucosidase*	Phloem-specific expression of dsRNA	*N*. *tabacum*	Decreased adult survival	Raza et al., 2016
*Glutathione S-transferase 5*	Phloem-specific expression of dsRNA	*Arabidopsis thaliana*	Prolonged nymph development	Eakteiman et al., 2018

Notably, phloem-specific expression of dsRNA that was achieved through the use of phloem-specific promoters, has shown high effectiveness in combating whiteflies. In tobacco plants, phloem-specific expression of dsRNA targeting two genes responsible for whitefly osmotic pressure maintenance led to a significant increase in whitefly mortality (Raza et al., 2016). Similarly, in *A. thaliana*, phloem-specific expression of dsRNA targeting whitefly detoxification genes extended the developmental period of whitefly nymphs (Eakteiman et al., 2018).

Manipulation of the expression of intrinsic or artificial micro RNAs (miRNAs) that target whitefly genes in plants was also shown to confer plants with whitefly resistance. Using *in silico* prediction, a cotton miRNA ghr-miR166b was found to target several whitefly genes involved in mitochondrial ATP synthase. Overexpression of ghr-miR166b in cotton plants significantly increased whitefly mortality and protected plants from whitefly infestation (Wamiq and Khan, 2018). Overexpression of an engineered artificial miRNA targeting three whitefly genes including *sex lethal*, *acetylcholinesterase* and *orcokinin* conferred high resistance against whitefly in tobacco plants (Zubair et al., 2020).

Furthermore, different strategies to generate transgenic plants that express dsRNA have been explored and compared. Currently, there are two main approaches for transforming, namely nuclear transformation and transplastomics (Zhang et al., 2017). Dong et al. (2020) found that transgenic tobacco plants derived from nuclear transformation were more effective for whitefly management than those derived from transplastomics. Further analysis revealed that the lower effectiveness of transplastomic plants could be attributed to the inability of whitefly to ingest dsRNA from plastids. This study not only highlights the difference between whitefly and insects with chewing mouthparts, but also provides reference information for optimizing RNAi-based resistance breeding against whitefly.

#### 5.2.2 Ectopic expression of foreign genes

The commonly-used Bt toxins are ineffective against hemipteran insects like whitefly due to their mode of action (Palma et al., 2014). Therefore, in resistance breeding against whitefly, insecticidal proteins other than Bt have been identified from various sources and utilized for ectopic expression. For example, ectopic expression of the *Aspergillus niger β-glucosidase* gene in tobacco plants markedly increased resistance against whitefly (Wei et al., 2007). In tobacco plants, expression of *Pinellia ternate* agglutinin, a protein with lectin and insecticidal activity against whitefly, resulted in a reduction of over 90% in whitefly nymphal survival and population size (Jin et al., 2012). Another study screened proteins from ferns, a group of plants known for their intrinsic resistance to whitefly, and identified Tma12, a protein with chitin-binding and chitinase activity from *Tectaria macrodonta*. Expression of Tma12 in cotton plants resulted in a reduction of over 90% in whitefly population, decreased the incidence of whitefly-borne cotton leaf curl viral diseases, and disrupted whitefly life cycles (Shukla et al., 2016). Additionally, tissue-specific expression of insecticidal proteins has been explored. Phloem-specific expression of a neurotoxin and an onion leaf lectin in tobacco plants resulted in nearly 100% whitefly mortality (Javaid et al., 2016). Moreover, effective resistance against both whitefly and cotton bollworm were achieved in cotton plants by expressing both Bt and *Allium sativum* lectin genes (Din et al., 2021). These studies demonstrate that while classical Bt toxins are not effective against whitefly, other insecticidal proteins from diverse sources may hold significant potential in managing whitefly and can be used in conjunction with Bt toxins to control multiple insect pests.

Another strategy for resistance breeding against whitefly involves manipulating the production of plant chemicals that display insecticidal properties. Some chemicals derived from plants have been found to be highly toxic to whitefly. Manipulating the production of these chemicals in plants can be achieved through the ectopic expression of foreign genes. For example, the over-expression of the *pectin methylesterase* gene from *A. thaliana* and *A. niger* in transgenic tobacco plants substantially increased methanol production, resulting in reduction of the whitefly population (Dixit et al., 2013). Ectopic expression of the *7-epizingiberene synthase* and *Z-Z-farnesyl-diphosphate synthase* genes from *S. habrochaites* in the glandular trichomes of *S. lycopersicum* plants led to the production of 7-epizingiberene, a chemical with toxic and repellent properties against whitefly (Bleeker et al., 2012).

## 6 Future perspectives

In the study and engineering of plant resistance against whitefly, significant progress has been made, but there are still important issues that require further exploration. One such question is the identification of whitefly-derived factors that trigger plant defense responses during whitefly herbivory. Another key area is the improvement of identification and utilization of plant resistance genes, thereby developing more effective breeding strategies. Additionally, the potential use of whitefly horizontally transferred genes (HTGs) as targets for RNAi in resistance breeding is worth investigating. Utilizing HTGs as RNAi targets could potentially enhance the efficacy and specificity of resistance breeding. These areas of research hold great promises in advancing our understanding of plant resistance to whitefly and developing innovative approaches for whitefly management.

### 6.1 Whitefly-derived elicitors of plant defenses

While physical traits of plant resistances are often expressed constitutively, chemical traits are often induced by whitefly herbivory. In the research to unravel the induction of chemical defense, mechanical damage and elicitors from saliva and eggs were found to mediate the perception of chewing insects by plants (Bonaventure, 2012). For phloem-feeders, a cysteine protease Cathepsin B3 from the saliva of aphids and a salivary protein NlG14 from the rice brown planthopper were shown to serve as elicitors of plant defense responses (Guo et al., 2020; Gao et al., 2022). Whiteflies exhibit distinct behavior and physiology compared to chewing insects and other phloem-feeding insects (Kempema et al., 2007; Walling, 2008). Consequently, the perception of whitefly feeding by plants may differ from that of the other insect herbivores. So far only one case study reported the activation of plant defenses by factors from whitefly. Whitefly may glycosylate salicylic acid ingested from plants and the secretion of honeydew containing salicylic acid glycoside may induce the accumulation of endogenous free salicylic acid and the expression of downstream genes in the salicylic acid signaling pathway (VanDoorn et al., 2015). Therefore, further investigations are necessary to identify whitefly-derived factors that mediate plant perception of whitefly herbivory. These factors could be metabolites or proteins that come into contact with plants during whitefly feeding, oviposition or honeydew secretion. Additionally, whitefly-derived nucleotides, such as small RNAs, have been shown to be transferred into plants during feeding, and may serve as potential elicitors of plants defenses (van Kleeff et al., 2016). Future studies in this area can draw upon research on the other groups of insect herbivores and harness sophisticated techniques including transgenes and RNAi.

### 6.2 Identification of genetic resources for resistance breeding

Few resistance genes from crops or their close wild relatives have been identified as possible genetic resources in resistance breeding against whitefly, and many of these genes have shown limited effectiveness (as mentioned earlier). Therefore, further efforts are needed to explore genetic resources from these plants. Additionally, it is worth considering alternative sources of genetic resistance. Resistance genes have already been discovered in unexpected sources. For example, Tma12 identified from fern exhibits high resistance to insect herbivores, including whitefly (Shukla et al., 2016). This suggests that resistance genes may be obtained from non-hosts or poor hosts of whitefly. Identification of these plants is relatively straightforward, and various strategies such as mass spectrum identification of insecticidal proteins, distant hybridization and genome-wide association studies can be utilized to identify key genomic loci associated with resistance. By exploring these diverse genetic resources, we can potentially uncover novel resistance genes for effective whitefly management.

### 6.3 Horizontally transferred genes as RNAi targets in resistance breeding

HTGs are acquired by organisms from other organisms through means other than reproduction. In whiteflies, dozens of HTGs have been discovered since their initial report in 2020 (Lapadula et al., 2020; Xia et al., 2021; Gilbert and Maumus, 2022; Li et al., 2022). Many of these HTGs appear to play important roles in the life history of whiteflies. For example, the HTG *BtPMaT1* from plants enables whiteflies to neutralize phenolic glucosides and feed on toxic plants (Xia et al., 2021). HTGs have unique biological importance in whiteflies and the presumably low prevalence of whitefly HTGs in other groups of insects make them ideal targets for RNAi. Recently, two studies targeting whitefly HGTs revealed that they can be used as targets in whitefly control without adverse effects on non-target organisms (Xia et al., 2021; Feng et al., 2023). This progress highlights the need for further exploration of HTGs as RNAi targets in resistance breeding. Additionally, the utilization of phloem-specific promoters can enhance the efficacy and specificity of RNAi technology. By harnessing HTGs and incorporating phloem-specific promoters, researchers can develop more effective and targeted approaches to combat whitefly infestation.

## Author contributions

L-LP and S-SL contributed to conception and design of this review. DL, H-YL, and J-RZ collected the references. DL, H-YL, J-RZ, Y-JW, and S-XZ wrote the first of the manuscript, and S-SL and L-LP revised the manuscript. All authors contributed to manuscript revision, read, and approved the submitted version.
